# Transmission Media of Foodborne Diseases as an Index Prediction of Diarrheagenic *Escherichia coli*: Study at Elementary School, Surabaya, Indonesia

**DOI:** 10.3390/ijerph17218227

**Published:** 2020-11-07

**Authors:** Fariani Syahrul, Chatarina U. Wahyuni, Hari B. Notobroto, Eddy B. Wasito, Annis C. Adi, Febi Dwirahmadi

**Affiliations:** 1 Department of Epidemiology, Faculty of Public Health, Universitas Airlangga, Surabaya 60115, Indonesia; chatrin03@yahoo.com; 2 Department of Biostatistic, Faculty of Public Health Universitas Airlangga, Surabaya 60115, Indonesia; haribasuki.n@fkm.unair.ac.id; 3 Department of Microbiology, Faculty of Medicine Universitas Airlangga, Surabaya 60115, Indonesia; eddybaguswasito@yahoo.co.id; 4 Department of Nutrition, Faculty of Public Health Universitas Airlangga, Surabaya 60115, Indonesia; annis_catur@fkm.unair.ac.id; 5 Center for Environment and Population Health, School of Medicine, Griffith University, Queensland 4215, Australia; f.dwirahmadi@griffith.edu.au

**Keywords:** children, diarrheagenic *Escherichia Coli*, foodborne diseases

## Abstract

Foodborne diseases (FBDs) have a large disease burden among children. The major type of FBD in children is diarrhea, caused mainly by contaminated food. One of the diarrhea pathogens is Diarrheagenic *Escherichia coli* (DEC). The aim of this study was to establish a model of microbial prediction (DEC) in stool, caused by the transmission of FBDs in elementary schoolchildren. An observational analytic study was conducted, with a nested case-control study design. In Stage I, the study population was children in a selected elementary school at Surabaya. The sample size for Stage I was 218 children. In Stage II, the case sample was all children with a positive test for DEC (15 children), and the control sample was all children who had tested negative for DEC (60 children). The result of the laboratory tests showed that the proportion of DEC in children was 6.88% (15 of 218 children) and the proportion of *Escherichia coli* O157:H7 in children was only 0.46%. The most significant mode of transmission included in the model was the snacking frequency at school and the risk classification of food that was often purchased at school. The formulation of the predicting model of DEC in stool can be used as an early warning against the incidence of FBDs in elementary schoolchildren.

## 1. Introduction

Foodborne diseases (FBDs) are one of the leading causes of morbidity and mortality worldwide, particularly among children in developing countries [1,2,3]. The World Health Organization (WHO) estimated that, every year around the world, the consumption of unsafe food causes 600 million cases of FBDs and 420,000 deaths. Every year, more than 150 million people are affected by FBDs, and more than 170,000 die. Diarrheal diseases are responsible for the majority of deaths because of FBDs in Southeast Asia [4].

FBDs are diseases caused by consuming contaminated food/drinks that contain various harmful microorganisms and pathogens. Various studies have found that there are three types of FBDs: enteric, parasitic, and chemical [5]. The major FBD in children is diarrhea, caused mainly by contaminated food. The potential underlying factors of FBDs among children are those related to behavior, including personal hygiene and poor snacking habits in school. In a previous study, Syahrul et al. suggested that snacking is very popular among schoolchildren and very difficult to change because students need to have food while at school. The results of the study in Surabaya showed that only 4.2% of students did not have a habit of snacking at school. This means that most students do not bring enough food from home [6]. In general, they only bring enough food to be consumed at one time.

The most common causes of FBDs were diarrheal disease agents, particularly Diarrheagenic *Escherichia coli* (DEC). DEC is common in various regions in the world. It depends on the host factor and its environment. *Escherichia coli* (*E. coli*) lives in human intestines and usually does not harm human health (it is a general bacterium). However, few *E. coli* strains obtain virulence genes through genetic horizontal transfer and cause gastroenteritis. DEC is the most important cause of acute gastroenteritis in children. However, until now, DEC-related epidemiology information has been limited [7]. DEC strains can be classified into six groups based on their clinical, epidemiological, and virulence properties: Enterohemorrhagic *E. coli* (EHEC), Enteropathogenic *E. coli* (EPEC), Enteroaggregative *E. coli* (EAEC), Enteroinvasive *E. coli* (EIEC), Diffusely adherent *E. coli* (DAEC), and Enterotoxigenic *E. coli* (ETEC) [8,9]. *E. coli* (Gram-negative bacteria) in the urinary tract are the most common cause of Urinary Tract Infections (UTIs), in both the community and nosocomial with *E. coli* being considered the main etiological agent. UTIs are infections of global concern [10,11].

Diarrhea caused by DEC infection remains a major public health issue, especially among children in developing countries [12]. Several research results show that annual diarrhea cases in children are quite high (3.2–4.6 episode), with the cause being contaminated food 70% of the time. However, the number of cases of FBD is under-reported [13].

In 2015, the incidence of diarrhea (a type of FBD) in all ages in Indonesia was 270 per 1000 population [14]. Studies conducted in several cities in Indonesia have found that more than 90% of FBDs were caused by microbiological contamination, including typhoid fever, bacterial/amoebic dysentery, botulism, and intoxication by other bacteria such as *listeriosis* and *trichinellosis* [15]. In 2015, in Sidoarjo, East Java Province, a study discovered that most sausages sold in the school environment contained *E. coli* and *Salmonella*. The same study also found that most rolled noodles also contained *E. coli*, making it unsafe for consumption [16].

Bacterial infection is one of the most important factors causing morbidity and mortality among infectious diseases, including *E. coli,* which is a Gram-negative bacteria. Several strains of *E. coli* live in human intestines and rarely cause diseases, but there are a number of *E. coli* strain pathogens that can cause diarrhea or extraintestinal diseases, in both healthy individuals and those whose immune system is disrupted. Various subtypes of Entero-virulent *E. coli* (EEC) strains are the main causes of diarrheal disease [17]. *E. coli* is a currently known pathotype, which is collectively known as Diarrheagenic *E. coli* (DEC). The pathotypes of DEC differ with respect to preferential host colonization location, virulence mechanism, and clinical symptoms and subsequent consequences, and are classified as EPEC, EHEC/STEC, EAEC, ETEC, and EIEC [12]. Several EPEC strains (atypical EPEC) are commonly found in animals. Interestingly some of these strains have been discovered related to diarrheal diseases in human (i.e., O26, O103, O119, O128, O142, and O157). Serotyping, multilocus sequence typing (MLST), and pulsed field gel electrophoresis (PFGE) have been proven effective to demonstrate that domestic animals (i.e., cat and dog), cattle (cow, sheep, and rabbit), and wild animals (monkey) as well as the environment are the potential sources of aEPEC transmission to human. These animals are the reservoir of atypical EPEC and serve as a source of human infection. Humans also have the potential to be EPEC reservoirs and could infect their domesticated animals. The transmission dynamic between reservoirs, however, remains unclear. In addition to this, food including raw meat, pasteurized milk, vegetables, and unsterilized water has also been reported to be able to transmit aEPEC to human [12,18]. Besides these wild animals, wild boar can act as carriers for various strains of pathogenic and anti-microbial resistant E. coli. The spread of these pathogenic and antimicrobial resistant *E. coli* to humans is probably due to the contact of these animals with domestic animals in extensive or semi-extensive livestock areas [19]. Thus, a collaborative One Health approach is urgently required to achieve optimal health outcomes for humans, animals, and the environment. Regarding this, Osawa et al. (2013) conducted a study using the microbial culture method to investigate the presence of DEC agents in both diarrhea-suspected children and non-diarrhea-suspected children in Surabaya, East Java Province, Indonesia [20]. Through this study, they confirmed the presence of DEC agents in both groups. Shiga toxin-producing *E. coli* (STEC), EPEC, EAEC, and EIEC were found in the non-suspect group, while EPEC, EAEC, ETEC, and EIEC were detected in the suspect group.

Imdad et al. confirmed that DEC agents are the main pathogens that cause diarrhea-related morbidity and mortality among infants and children [7]; however, little is known regarding DEC prediction models using stool by employing FBD transmission media variables. Therefore, this study aimed to establish a DEC prediction model through the stool culture method among elementary school-aged children in the city of Surabaya. This study will be useful in informing relevant stakeholders on how to predict DEC presence among children and how to develop effective health promotion measures in the context of elementary school-aged children, not only in Surabaya, but also in other cities of Indonesia.

## 2. Materials and Methods

### 2.1. Study Design

This study adopted an observational analytic approach and had a nested case-control design. It was conducted across state elementary schools in two sub-districts, Sawahan and Semampir, in Surabaya City. The site selection was based on the diarrhea case data of elementary school-aged children (6–12 years old) from the Health Office of Surabaya City. Two elementary schools were selected from each sub-district; thus, there were four schools in total. The data were collected over eight months (from December 2016 to July 2017) and have been validated.

### 2.2. Participants

In Stage I, the research population of the cohort group was all children of the chosen state elementary school, with the criteria that the school had a canteen and food/drink street vendors around the school. This study applied inclusion and exclusion criteria to ensure that the samples were relevant to the objective of the study.

Inclusion criteria were: (a) during data collection, the children were from the fourth and fifth grades because, during this age, children start cultivating snacking habits in school and start to rarely bring lunch from home [21,22]; (b) the children’s residential addresses were in the same sub-district as their school so that the condition of the food/drink offered by the street vendors around the residence and school would be relatively the same; and (c) the parents of the children agreed to sign an informed consent form.

Exclusion criteria were: (a) the nutritional status of the children was very thin or thin (nutritional status was measured using anthropometry method with IMT/U index); (b) drank yogurt and/or fermented milk in the last seven days before data collection, with at least four servings, as yogurt and fermented milk contain probiotic bacteria (such as *Lactobacillus*), which can increase the immune response of the digestive tract and maintains body health [23], and the period was taken as seven days because the period needed for food to enter the esophagus and reach the rectum is 3–7 days [24]; and (c) the children were consuming antibiotic medication.

**Figure 1:**
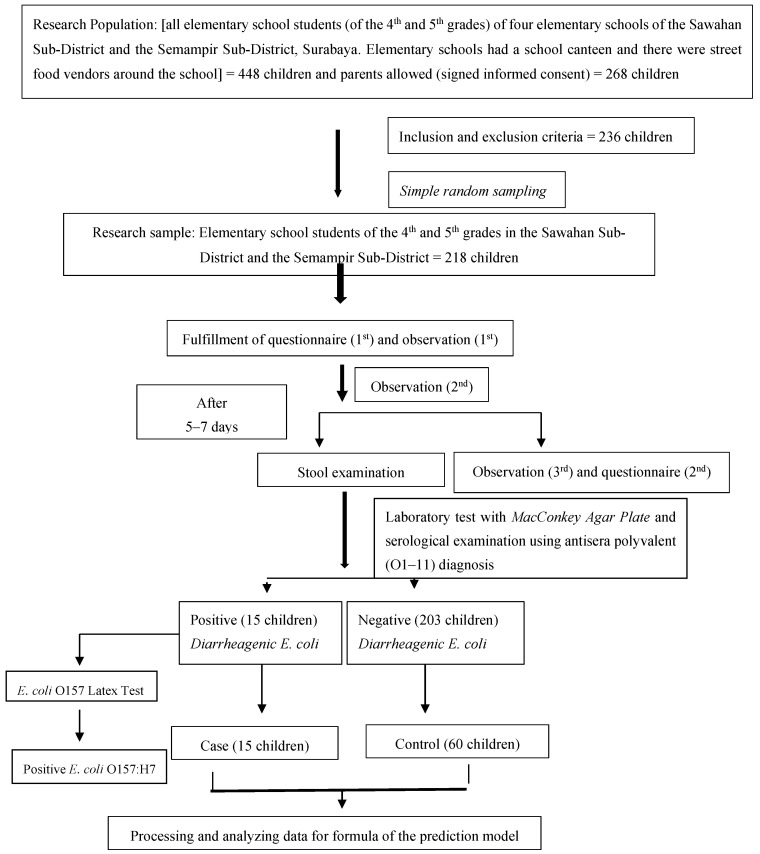
Flow of the research process.

In the second stage, the result (outcome) of the related variable measurement in the first stage had two conditions, which were children identified as having DEC and children identified as not having DEC in their stools. Therefore, in the second stage, the children who were identified as being positive for the presence of DEC in their stools were the case sample, while children who were identified as being negative for the presence of DEC in their stools were the control sample. The flow of the research process can be seen in Figure 1.

### 2.3. Research Question

Can the transmission media of FBDs be used as an index prediction for DEC?

### 2.4. Research Variable and Data Collection

The dependent variable was the presence of DEC as an outcome. Meanwhile, the exposure variables were handwashing habits, fingernail hygiene, snacking frequencies at school, snacking frequencies at home, bringing lunch to school, type of food/beverage consumed, food risk classification, and snacking location. The Potential Confounding Variables (PCVs) in this research were nutritional status, antibiotic usage, and yogurt and/or fermented milk consumption (containing probiotics). The PCVs were controlled by performing restrictions.

The data collection and DEC presence measurement method in stool were performed by providing plastic pots for the children, including equipment to contain the stool during defecation at home. Each pot was given a code and labeled with the child’s name to minimize the possibility of being exchanged with other children’s stool samples. Laboratory tests were conducted on the stool samples to identify the presence of DEC, which were obtained by using the MacConkey agar media method and followed by a serological examination using an O1–11 polyvalent (Bio Farma Bandung, Indonesia) antisera diagnosis. *E. coli* O157:H7 was detected using an *E. coli* O157 Latex Test (Oxoid brand). The laboratory test was conducted in the microbiology laboratory of the Institute of Tropical Disease (ITD) of Universitas Airlangga. Other variables were measured using questionnaires fulfilled by the children, accompanied by enumeration and observation.

In this research, a possible bias that might have occurred is the contamination of *E. coli* in food/beverages during the material supply and production process. Therefore, the conditions of the food and beverages identified in this study were food/beverages that were circulating around the consumers and not during the process of supplying the materials and production. The study was conducted at state elementary schools in urban areas only; thus, there is a possibility that the results would differ from a similar study conducted on state elementary schools in rural areas.

Prior to the data collection activities of four elementary schools, permission was obtained from the National Unity, Politics and Community Protection Agency of Surabaya City, and the District Education Office with a cover letter from the Faculty of Public Health, Universitas Airlangga for an ethical clearance of the study. The ethical clearance approval was obtained from the Ethics Commission of the Faculty of Public Health, Universitas Airlangga, with Eligibility Certificate No. 627-KEPK.

### 2.5. Sample Size

In the first stage, the sample size used the large sample formula from Lemeshow et al. (1990) for a case-control study and corrected with a prevalence rate of 6.9% [6]. Through the calculation of the pathogenic bacteria prevalence as 6.9%, the sample size for the first stage was 15/6.9% = 217.39 ≈ 218 children. The sample size for the second stage was 1:4 for case-control; thus, there were 15 cases and 60 controls.

In the second stage, the sampling method for control cases was the following: (1) case sample was devised by including all children with positive laboratory test results for DEC, which was as many as 15 children; and (2) control samples were selected using simple random sampling from all children with negative laboratory test results of DEC, which were as many as 60 children (case sample:control sample comparison was 1:4). The control sample specimens were selected randomly but were from children at the same school as the case samples.

### 2.6. Statistical Analysis

For the formulation of a prediction model for the presence of DEC in stool through the transmission media of FBDs, the binomial logistic regression model was used, with a significance level of 0.05. The prediction model was formulated in the form of mathematical formulas.

## 3. Results

### 3.1. Characteristics of the Children

The results of the first phase of research show that the average age range was 8–13 years. The complete results can be seen in Table 1.

### 3.2. Proportion Rate of DEC in Children

The result of the laboratory test showed that the proportion rate of DEC in children was 6.88% (15 children from 218) and the proportion rate of *E. coli* O157:H7 in children was only 0.46%.

The result of the total plate count (TPC) in children’s stool that had tested positive for DEC was from 11 × 10^4^/mL to 132 × 10^4^/ mL. For children who had tested positive for *E. coli* O157:H7, the TPC was 110 × 10^4^/ mL. The infectious dose of DEC was very low with 1–10 bacteria for *E. coli* O157:H7 and 10–100 for bacteria other than *E. coli* O157:H7 [25,26,27].

Children who were detected as being positive of having DEC qualified as the case sample for the second stage, while as many as 60 children who had tested negative for DEC were selected through random sampling to become the control sample.

### 3.3. Fingernail Hygiene

The results show the conditions of the fingernails of the children in the case group. Most of them had poor fingernail hygiene (60.0%), while children in the control group mostly had good fingernail conditions, that is, their nails were kept short and clean. The complete results can be seen in Table 2.

### 3.4. Handwashing Habits at School

The results show the handwashing habits practiced at school; most of the children in the case and control groups had poor handwashing habits at school. The complete results can be seen in Table 3.

### 3.5. The Habit of Consuming Snacks at School

The habit of consuming snacks at school included the habit of bringing lunch from home, the snacking frequency at school, risk classification of the food that was often purchased at school, and snack location.

### 3.6. The Habit of Bringing Lunch from Home

The results show that the majority of children in the case group (46.7%) were in the habit of bringing lunch to school, and, in the control group, 70.0% stated that they sometimes packed lunch for school. The complete results can be seen in Table 4.

### 3.7. Snacking Frequency at School

The results show that most children (66.7%) who tested positive for DEC in their stool had a snacking frequency of ≥4 times/week. Meanwhile, children who tested negative for DEC (66.7%) had a snacking frequency of mostly 1–3 times/week. The complete results can be seen in Table 5.

### 3.8. Snacking Location

The snacking locations at school were street food stalls, stalls of food sellers outside the school, and the canteen inside the school. Meanwhile, the snacking locations at home were street food stalls, food shops near the house, restaurants, and mini-markets.

### 3.9. Food Risk Classification

The food and/or beverages assessed were those that were often bought by children at school and those that were bought during the questionnaire fulfillment and observation. Food and beverages categorized as high risk were food and beverages that have a high potential of being contaminated by the *E. coli* bacteria (Table 6).

The results indicate that there were many children who bought food and/or beverages with a high risk of being contaminated with *E. Coli* (Table 7).

### 3.10. Formulation of the Prediction Model for the Presence of DEC Bacteria in Stool through Transmission Media in Children

The results of the logistic regression analysis demonstrate that only the snacking frequency variable and the snacking condition can be included in the prediction model formulation (Table 8).

Probability formulation obtained is as follows:

(1) $$Prob\;\left(Y=1\right)=\frac{1}{\;1+e-\left(-2.734+1.502\;X1+1.307\;X2\right)}$$

where *X*1 is the snacking frequency (code 1 = ≥ 4 times/week) and *X*2 is the food risk classification (Code 1 = high risk). A goodness of fit score of 0.659 means that the model produced is fit.

Table 9 presents the simulation result of probability formulation.

### 3.11. Limitations of the Study

The food and beverages identified as being likely to be contaminated with DEC are the food and beverages sold in schools and around schools, and the condition of these from their time of production and the materials used in its production was not checked.

## 4. Discussion

*E. coli* is an opportunistic pathogen. When it is in a certain place of the human body, it can cause health problems ranging from mild stomach pain to diarrhea, UTIs, sepsis, and meningitis [29]. *E. coli* is the main cause of UTIs, and uncomplicated UTI infection is mostly caused by *E. coli* [30,31]. This study found DEC in children’s stool. According to theory, the bacteria found can be classified as EPEC, which is a bacterium that often causes diarrhea in infants. ETEC is a bacterium responsible for diarrhea in infants and travelers, while EHEC is a bacterium associated with hemorrhagic colitis and hemolytic uremic syndromes. EIEC is a bacterium that causes shigellosis, and EAEC is a bacterium that causes acute diarrhea and chronic diarrhea [29,32]. One strain of DEC that has high virulence is *E. coli* O157:H7, which once caused an outbreak in the United States [29]. The identification of DEC, especially EHEC, was conducted using a MacConkey agar plate, followed by a serological examination using polyvalent diagnostic antisera.

The proportion of children who tested positive for the presence of DEC in their stools was 6.88%, while the proportion of children who tested positive for *E. coli* O157: H7 in their stool was only 0.46%. DEC bacteria are one of the causes of FBDs, including diarrhea. Indeed, although the proportions are small, the virulence level of the DEC bacteria is quite high. In certain strains, such as EHEC and EAEC, it can cause hemolytic uremia syndrome, kidney failure, meningitis, and bacteremia [33]. The proportion of 6.88% found in this study is lower than the one in the study conducted by Nakasone et al. who calculated that the prevalence of DEC in primary school-aged children in Surabaya in 1995 was 11.6%. By looking at these two numbers, it can be stated that the positive presence of DEC in the stool of school-aged children of 12 years has decreased, but it has taken quite a long time (from 1995 to 2017). In the study conducted by Nakasone et al. in 1995, the prevalence rate found was also known as the carrier rate.

DEC is one of the agents of FBDs. The reduction of FBD cases is one of the main objectives of national and international food safety programs [34]. Variables that can be used to predict the presence of DEC in children’s stool are children’s snacking frequency at school and the condition of snacks bought at school. The prediction model for DEC, created using the knowledge of the various transmission media of DEC in elementary school-aged children and in the form of mathematical formulas, can be used to predict the presence of DEC in children by observing the frequency of their snacking and the risk classification of food that is often bought at school.

The frequency of having snacks at school is one of the variables that can be used to predict the presence of DEC in schoolchildren’s stool. This is in accordance with one of the causal relationship theories, namely, the dose–response relationship, in which the higher is the dose of exposure, the greater is the chance of an outcome. In this study, the greater is the snacking frequency of a child at school, the greater is chance of the child having DEC in their stools. A child whose snacking frequency is four times per week has a higher chance of consuming food and/or beverages snacks contaminated with microbes, especially *E. coli*. The results of the identification of food and drink snacks show that, in all locations selling snacks in all schools, there were foods and beverages that could have been contaminated with *E. coli*.

The variable of risk classification of food that is bought at school has an OR value of 3.696 with a value of 95% CI: 1.071 < OR < 12.758, which means that the risk of stool being detected DEC-positive is 3.696 times greater for children who often buy snacks with a higher risk than children who buy low-risk snacks. Here, snacks mean food and/or beverages bought by children at school. Food and beverages are categorized as high risk if they have a high chance of being contaminated by microbes, especially *E. coli* bacteria. It is well known that *E. coli* is often present in meat that has not been cooked properly, drinking water that has not been purified properly, or food that has been cross-contaminated from food handlers. The results of the analytic studies in the foodservice units at schools conclude that washing and food safety by food handlers had not been properly addressed [35]. Handling food (from production to storage) that is not safe can contribute to the risk of getting FBDs [36,37]. Therefore, it is necessary to increase the knowledge and awareness of schoolchildren regarding FBD risks for long-term prevention. In addition, the government must conduct active monitoring and training on food security and FBDs [38].

Poor handwashing in schools is actually a risk factor for the presence of DEC bacteria in children’s stools. In this study, children’s handwashing habit has not been taken as a predictor variable for the presence of DEC bacteria in children’s stool. All snacks sold around the school were accepted by children with individual packaging, both individually packaged since its production and when snacks were given to children. Individual packaging material included brown oiled paper, white paper, plastic wrapping, mica, and stick skewers. In one of the school canteens, snacks were being served in an open condition, so they could have been easily polluted by dust and flies. On the table where the food was served, pieces of brown oil paper were provided to cover the base when the child took the food. Therefore, handwashing habits were not related to the presence of DEC in children’s stools, as the child’s hands were not in direct contact with the snacks.

This study uses the concept of a healthy paradigm. The resulting prediction model is able to estimate the presence of DEC in stools, before a child has diarrhea, which is included in FBDs. Therefore, it is expected that a child is prevented from being at risk of developing diarrhea due to DEC or can be said to minimize the risk factors for diarrhea in children. DEC not only causes diarrhea but can also attack parts of the body outside the digestive tract. The results of the study show that, even though most children brought packed lunch, they still bought food and/or drinks during the school break or after school.

The prediction model for the presence of DEC in stools through transmission media, namely, the frequency of snacks at school and the conditions of snacks that are often bought at this school, can be used as an “early warning” so that children can avoid getting FBDs. That is, it can reduce the number of FBDs, especially diarrhea in children who have a habit of snacking at school.

## 5. Conclusions

Most children who were DEC-positive bought snacks that were classified as high-risk food and beverages, while most children who were DEC-negative bought snacks that were classified as being low-risk food and beverages. The decisions of schoolchildren in choosing and buying food and beverages were not based on their quality and safety.

In conditions of moderate and poor handwashing behavior, the variables that can be used to predict the presence of DEC bacteria in children’s stool are the snacking frequency at school and the conditions of snacks bought at school.

This study recommends the following:

1. Schools should have the capacity to form or reactivate the School Food Safety Team (TKP) so that they can ensure that the food and beverages sold in the canteen and around the school are safe to consume.
2. Attention should be paid to children’s high frequency of snacking at school; it is recommended that parents try to prepare lunches for children to bring to school.
3. There is a need for guiding food vendors, including canteen managers, on maintaining the safety of food and beverage conditions, as well as paying attention to individual packaging material for snacks.
4. The prediction model for the presence of DEC bacteria in children’s stools can be used as an “early warning” for the occurrence of FBDs in elementary schoolchildren.
5. Further research is needed to ascertain the dynamics of transmission between reservoirs (humans and animals), in line with the goals of One World One Health.

## Figures and Tables

**Table 1:** Distribution of age and sex of the students in Surabaya, 2017.

Characteristics	*n*	%
Age (years)		
8–9	17	7.8
10–11	189	86.7
12–13	12	5.5
Total	218	100.0
Sex		
Boys	84	38.5
Girls	134	61.5
Total	218	100.0

**Table 2:** Distribution of the fingernail conditions of the students based on the presence of DEC in stool, in Surabaya, 2017.

Fingernail Conditions	Positive DEC		Negative DEC	
	*n*	%	*n*	%
Poor	9	60.0	21	35.0
Good *	6	40.0	39	65.0
Total	15	100.0	60	100.0

* Good = clean and short.

**Table 3:** Distribution of handwashing habits at school based on the presence of *DEC* in stool, in Surabaya, 2017.

Handwashing Habits at School	Positive DEC		Negative DEC	
	*n*	%	*n*	%
Poor	7	46.7	31	51.7
Medium	5	33.3	23	38.3
Good	3	20.0	6	10.0
Total	15	100.0	60	100.0

**Table 4:** Distribution of children who had the habit of bringing lunch from home based on the presence of DEC in their stool, in Surabaya, 2017.

The Habit of Bringing Lunch from Home	Positive DEC		Negative DEC	
	*n*	%	*n*	%
Never	5	33.3	15	25.0
1–3 times/week	7	46.7	42	70.0
≥4 times/week	3	20.0	3	5.0
Total	15	100.0	60	100.0

**Table 5:** Distribution of children’s snacking frequency based on the presence of DEC in their stool, in Surabaya, 2017.

Snacking Frequency	Positive DEC		Negative DEC	
	*n*	%	*n*	%
At school				
≥4 times/week	10	66.7	20	33.3
1–3 times/week	5	33.3	40	66.7
Total	15	100.0	60	100.0

**Table 6:** Food Risk Classification based on Contamination Risk by *E. coli* bacteria, in Surabaya, 2017.

Category of Risk	Food	Beverage
Low Risk	Packaged snacksh Food that was packaged by the producer (e.g., bread, donut, chips) Main food (e.g., fried rice, yellow rice, rice with fried chicken, and others)	Bottled beverages Warm beverages
High Risk	Food with peanut sauce, *petis*, or other types of sauce (e.g., *cilok, cireng*, meatball, *batagor* seasoning, meatball sauce, and others).	Cold beverages (iced tea, coconut ice, syrup ice, etc.)

Source: [15,28].

**Table 7:** Distribution of food risk classification in school based on DEC in stools, in Surabaya, 2017.

Food Risk Classification	Positive DEC		Negative DEC	
	*n*	%	*n*	%
FOOD				
High risk	10	66.7	21	35.0
Low risk	5	33.3	39	65.0
Total	15	100.0	60	100.0
BEVERAGE				
High risk	9	60.0	18	30.0
Low risk	6	40.0	42	70.0
Total	15	100.0	60	100.0

**Table 8:** Results of the logistic regression analysis.

Variable	B	df	Sig	OR	95% CI	
					Lower	Upper
Snacking Frequency at School (≥4 times/week)	1.502	1	0.019	4.492	1.278	15.787
Food Risk Classification (high risk)	1.307	1	0.039	3.696	1.071	12.758

**Table 9:** Probability of stool being DEC positive.

Snack Frequency	Food Risk Classification	Probability
(1) ≥ 4 times/week	(1) high risk	51.88%
(1) ≥ 4 times/week	(0) low risk	22.58%
(0) < 4 times/week	(1) high risk	19.36%
(0) < 4 times/week	(0) low risk	6.09%
